# Diffusion dynamics of technological innovations in health: the case of an innovative medical imaging technology in France

**DOI:** 10.3389/fmed.2026.1654025

**Published:** 2026-06-10

**Authors:** Renaud Guignard, Bénédicte Geffroy, Valérie Fernandez

**Affiliations:** 1 Scintidoc, Montpellier, France; 2 Laboratoire d'économie et de management de Nantes Atlantique (LEMNA), département interdisciplinaire de sciences sociales (DI2S) d'IMT Atlantique, Nantes, France; 3 Institut interdisciplinaire de l’innovation (i3 CNRS), Telecom Paris, Institut Polytechnique de Paris, Palaiseau, France

**Keywords:** diffusion of innovation, distributed governance, hybrid medical imaging, PET-CT, qualitative research

## Abstract

**Objectives:**

Reports from France’s Cour des Comptes highlight a lack of aggregate data on medical imaging use. This study aims to (1) map the diffusion of PET-CT technology in France (2013–2022) and (2) identify the multi-level contextual factors explaining its uneven diffusion and utilization.

**Methods:**

We employed a phased, mixed-methods design. First, we consolidated quantitative data from national databases to create aggregate proxy indicators for supply, demand, and procedures. Second, we conducted 39 semi-structured interviews with a stratified, purposive sample of key stakeholders (regulators, clinicians) and analyzed them using inductive thematic analysis.

**Results:**

The supply of PET-CT technology has expanded coherently nationwide. However, its actual use varies significantly between regions, a disparity not explained by supply or epidemiological demand. This ‘supply-use’ gap is driven by interconnected factors: institutional (decentralized/distributed governance, delayed recommendations), organizational (competing ‘research’ vs. ‘access’ models of care), and professional (individual physician beliefs, variable participation in MDTs).

**Conclusion:**

The diffusion of PET-CT in France challenges a purely rational model, going beyond simple supply–demand logic. It is a complex process where local institutional, organizational, and professional contexts shape trajectories. Understanding these determinants is urgent for developing equitable health policy and addressing public health challenges in nuclear medicine.

## 1 Introduction

At the turn of the 21st century, a major innovation was introduced in the field of medical imaging: PET-CT (Positron Emission Tomography – Computed Tomography). This new technology enabled more precise cancer diagnoses and treatment efficacy assessments. *Time* magazine crowned it ‘medical invention of the year’ in its 4 December 2000 issue, and the evidence-based medicine literature has highlighted both its cost-effectiveness and its efficacy in improving treatments for cancer patients.

Before discussing the specifics of the French context, it is crucial to situate this study within the broader academic literature on the diffusion of innovations in healthcare. Research in this field, building on foundational work in innovation studies, has moved beyond simple adoption curves to explore the complex interplay of technology, professional networks, organizational structures, and institutional policies (1–3). While Evidence-Based Medicine (EBM) aims for standardization (4), the actual implementation of new technologies is often uneven, reflecting “local universality” (5) rather than true standardization. Our work aims to contribute to this conversation by examining how these multi-level factors shape the diffusion of a mature, high-cost medical technology.

As Ferlie et al. (6), among others, have pointed out, the diffusion of medical innovations—the process by which a new technology is adopted by a growing number of players—is complex, controversial, and dependent on multiple contextual and non-technical factors. In recent years, the rise of evidence-based medicine (EBM) has led to the dissemination of “best practices” and helped to standardize medical approaches that use emerging technological innovations in the medical field (4).

The diffusion of valuable medical innovations is often assumed to be a rational process, yet it is rarely straightforward. Research has shown that even with clear clinical benefits and institutional supervision, substantial, non-rational differences in adoption persist between regions and practitioners (5, 7). Furthermore, drawing upon the theoretical framework of the sociology of technology, it is posited that users and technologies are ‘co-constructed’. This implies that technologies are frequently implicitly designed for and adopted by those who possess the most resources, access, and voice (8). This uneven diffusion is then not just a logistical problem; it raises urgent questions of health equity, as access disparities can widen, rather than close, existing socioeconomic health gaps. Finally, a significant gap persists in the literature between studies of *diffusion* (the “spread”) and studies of successful *dissemination* (the “impact” on patient health outcomes). The trajectory of PET-CT in France therefore provides a powerful case study of this very problem. Following initial state-supported investments (2003–2013), adoption has lagged behind what a purely rational model would predict. This leads us to ask: What is the actual situation regarding the dissemination of this technology and its trajectory? Our study addresses this by identifying the contextual factors of diffusion, providing the necessary foundation for the more urgent, outcome-focused inquiry.

The research presented here analyses the diffusion trajectory, in France, of an innovative technology in the field of medical imaging: PET-CT. It was carried out under a research programme on nuclear medicine applied to cancerology, which has been awarded a *LABel d’Excellence* grant (LABEx IRON), an internationally recognized distinction for research. This part of the research programme is based on two key reports on medical imaging, published by the *Cour des Comptes* (France’s main public policy assessment body) in 2016 and 2022. These reports highlight the lack of relevant information for analysing the spread of technologies in the sector and the regional disparities concerning them. The existence of “organised disorder” and “unexplained” regional inequalities in the heavy equipment authorisation system has been corroborated by academic research, highlighting a failure of the regulatory system that exacerbates inequalities in access (9). Our study takes stock of the national dynamics of PET-CT diffusion, with the primary objective of understanding how key factors interact in specific local contexts to produce different regional trajectories.

## 2 Materials and methods

We draw on two sets of original data, which we created in compliance with legal authorizations and the rules of scientific integrity. In order to statistically analyse data on the deployment and dissemination of the technology, a first set of discrete data covering the years 2013 to 2022 was collected from various reports by national organisations and open databases (e.g., the National Institute for Demographic Studies (INED), the Research, Studies, Evaluation and Statistics Directorate (DREES), or the epidemiological reports from the National Cancer Institute InCA), on equipment deployment, medical demographics, demographic and epidemiological data, and the volume of imaging procedures.

Following public policy analysis procedures for the field, we constructed synthetic indicators for macro analyses. One of these was an aggregate indicator of medical and technical resources (the number of PET cameras and the number of active nuclear medicine practitioners) reflecting care supply at the national and regional level; another indicator reflected the demand for medical imaging (number of cancer patients entering the care pathway, a variable linked to demographic and epidemiological data) at these same two levels of analysis. We acknowledge that this aggregate indicator serves as a proxy for demand and does not capture the heterogeneous imaging needs associated with different cancer types; however, it is the most consistent longitudinal data available for this macro-level analysis. We also gathered data on numbers of PET-CT imaging procedures performed (from the French Society of Nuclear medicine (SFMN) annual surveys), which provided an overview of the technology’s diffusion. This initial set of quantitative data and aggregated determinants allowed us to draw up a diffusion curve for PET-CT technology in France and in the various French regions between 2013 and 2022.

To better understand the dynamics of dissemination at the national level and the regional disparities observed, we combined our quantitative approach with a qualitative research approach. We drew a second set of qualitative data from a series of semi-structured interviews (*n* = 39) with a purposive sample of key players in the dissemination of medical imaging technologies: regional regulatory agencies, members of standards committees, doctors in charge of imaging centers, nuclear medicine (NM) practitioners, oncologists. This sample was stratified to ensure representation across different roles and geographic regions (including both high- and low-utilization areas) to capture a diversity of perspectives. The interview guide was developed based on a review of innovation diffusion literature and iteratively refined. All interviews were transcribed and coded using thematic analysis in NVivo. The analysis followed an inductive approach, where themes were derived directly from the data by three researchers, with disagreements resolved through consensus to ensure reliability. With the intention of leading them as close as possible to the subject of factors explaining the spread of PET-CT, we asked broad questions about innovations in medical imaging, the way they are regulated, the innovative aspects of PET-CT, the factors driving adoption and barriers to use, clinical practices, and related topics.

## 3 Results

**Figure 1:**
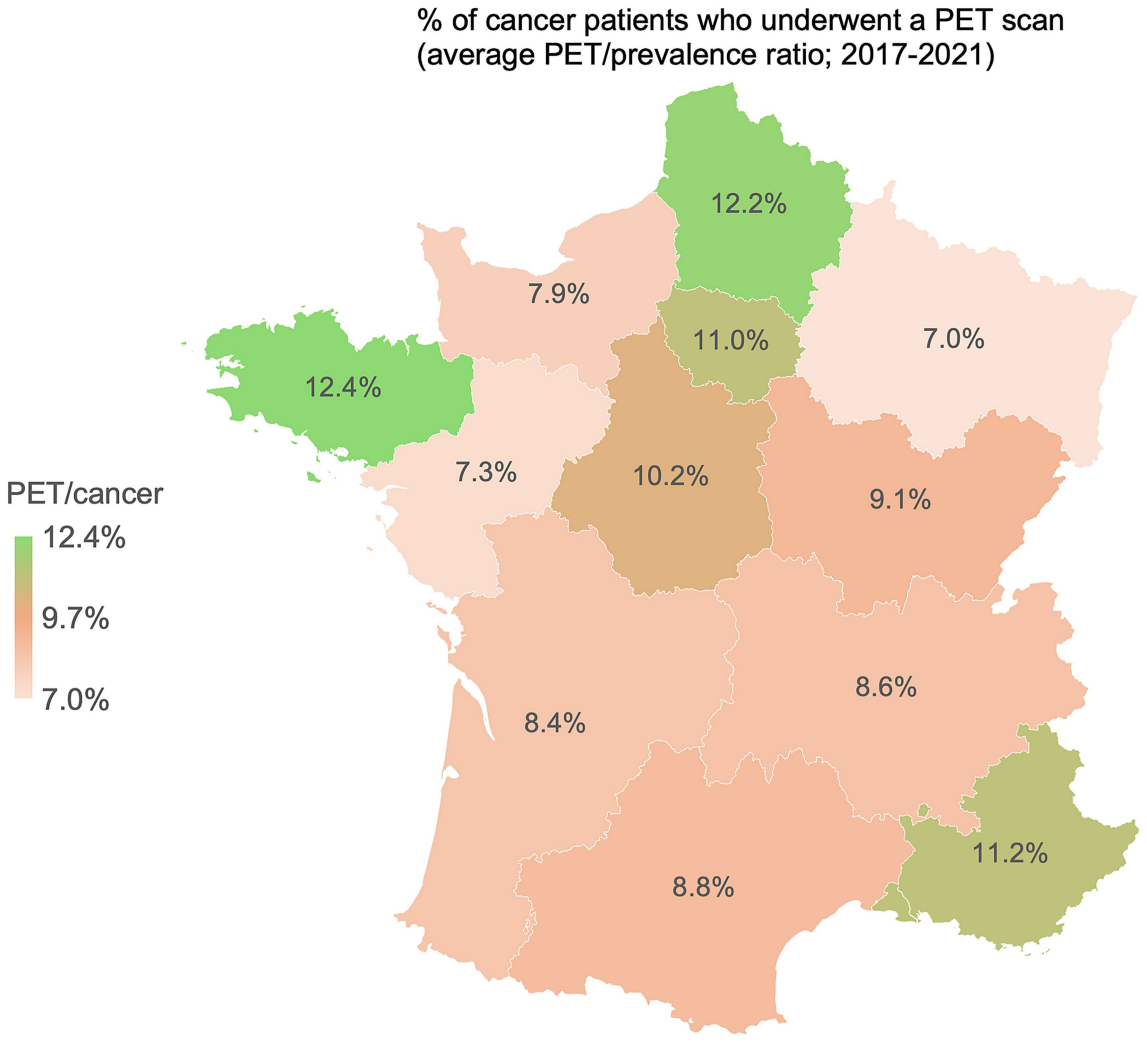
Percentage of cancer patients who underwent a PET scan (average PET/prevalence ratio; period: 2017–2021). The denominator (true prevalence) represents the total number of cancer patients over the given period. This metric serves as a proxy for technology utilization relative to actual demand.

**Figure 2:**
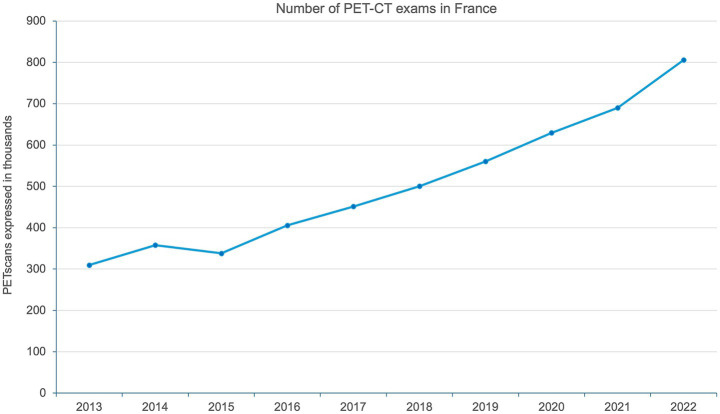
Number of PET-CT exams performed annually in France (period: 2013–2022; data source: annual surveys from the French Society of Nuclear Medicine, SFMN).

In this section, we present only aggregated data in the form of curves (Figure 1), maps (Figure 2) and tables (Table 1). The main significant data collected on the deployment and dissemination of PET-CT in all French regions are presented as Supplementary files.

**Table 1:** Main determinants of PET-CT regional dissemination.

Institutional and regulatory factors	Role of the Regional Health Agencies (ARS) in allocating equipment. Production and distribution of medical recommendations.
Organizational and structural factors	Care provision models (clinical vs. research) influencing the availability of equipment. Differences in organization between institutions (schedules, prioritization of examinations, waiting time). Shortage of medical and paramedical staff hindering the optimization of available resources.
Professional and individual factors	Differences in clinicians’ adoption of the technology depending on their perception of it. Role of multidisciplinary team meetings in disseminating the practice. Influence of opinion leaders and individual resistance to the adoption of PET-CT.

### 3.1 Nationwide diffusion

Our data on the number of PET-CT exams enables us to plot the technology’s diffusion curve between 2013 and 2022 (Figure 1). This curve shows steady, even exponential growth from 2015 onwards. At a macro level, this S-shaped trajectory appears to follow a classic adoption pattern. However, as the following sections demonstrate, this national average mask significant regional variations and complex underlying dynamics that challenge a purely rational model of diffusion based solely on clinical and cost-effectiveness values.

### 3.2 Regional diffusion

Notwithstanding certain region-dependent time lags in the deployment of equipment and associated human resources, our data show a coherent supply across the country, both in terms of the amount of equipment and human resources, and in terms of the spatial distribution of the offer in urban and peri-urban areas. However, they point to regional disparities in the use of the technology, that is, camera usage rates (ratio of number of examinations/camera/year), which reflect neither the supply of care (equipment and staff resources) nor the demand (patients entering a care pathway). Our findings thus show that the abundance of equipment does not guarantee that it will be used; actual activity may fall short of the supply of care (and the demand).

In processing our data, we were careful to control for the socio-demographic variables of the regions and for factors specific to the patient population, namely the epidemiological characteristics (varying prevalence of cancer across regions). The map in Figure 2 summarizes our analysis based on an aggregate determinant: the percentage of cancer patients who underwent a PET scan. The data highlight regional differences in the spread of PET-CT. They do not explain the mechanisms underlying these disparities.

### 3.3 Understanding the mechanisms of diffusion

We supplemented our approach with a qualitative inquiry to identify the various factors influencing regional dissemination dynamics and their differences. Interviews with representatives of a wide range of categories of players involved in the process of disseminating PET-CT have brought to light a set of factors that explain this process. Main determinants identified in our analysis are reported below and summarized in Table 1.

#### 3.3.1 Institutional and regulatory factors

In the French model, decisions regarding the allocation of medical imaging equipment are decentralized and entrusted to each region’s ARS (Agence Régionale de Santé – regional healthcare agency). In theory, this should allow the distribution of this equipment to be tailored to each region’s specific needs. Our results show that this system does not always guarantee optimal resource allocation and can lead to regional disparities. According to one ARS manager we interviewed, this system of delegating authorization does not really allow for the distribution of equipment across the country to be steered in such a way as to ensure equitable access to healthcare. This is because installing machines is not sufficient, by itself, to guarantee that they will actually be available to use.

Another institutional factor at play in countrywide dissemination is the production and distribution of medical recommendations on the proper use of PET-CT in oncology (10). This is a long and complex process. By the time these recommendations on the proper use of the technology are finally published, the innovation has already spread through other channels. The role of these recommendations, then, is no longer to promote the adoption of the technology, but rather to validate practices that have become commonplace.

As one of the writers of these recommendations pointed out in an interview: ‘*Most of the time, they tend to validate well-established knowledge rather than offer truly up-to-date information on the situation. So, at worst, they still give us standardized care, basic but not innovative*’. Ultimately, their impact is not to promote the immediate adoption of a recent innovation and drive rapid changes in medical practice. Rather, they serve to consolidate a consensus based on evidence and accumulated experience.

#### 3.3.2 Organizational and structural factors

On the level of individual facilities, our analysis of our interviews highlights illustrative examples of different ‘models of care provision’ defined within clinics and hospitals. Given identical resources (equipment and staff), wards have leeway to manage the availability of diagnostic resources and the allocation of specialist staff according to their consultation schedules. These discretionary choices can be explained by a ward’s choice to be oriented towards research or towards clinical care. As an ARS manager explained: ‘*In region X, my colleague who was in charge of imaging told me about it. She said: “Oh, but they’re doing research, so they’re going to use it for that*”’.

In the case of a research-oriented model, atypical examinations are therefore more likely to be carried out, leading to a lower overall number of scans. Conversely, these examinations have a higher ‘research and innovation’ value. Other wards have opted to focus on access to care. As one head of a nuclear medicine department put it: ‘*The first advantage in obtaining an examination is the short waiting time. If I have a choice between a CT scan or a PET scan, if I can have the test done the next day, I’ll take the one I have the next day … our model has been to shorten waiting times as quickly as possible and, secondly, to provide the report less than 24 hours after the test was carried out, meaning the report had to be sent to the requester the next morning*’. There are therefore different supply models: those targeting excellence in research, and those aiming for accessibility. In some regions, this is compounded by human resources issues, especially with paramedical staff, which are an obstacle to the optimization of equipment use.

#### 3.3.3 Professional and individual factors

Despite the existence of national recommendations, adherence to medical guidelines remains inconsistent. This ties in with local complexities, contexts that may encourage or discourage their use to varying degrees. As one head of a nuclear medicine department pointed out, if a requesting doctor –particularly if they are a department’s chief physician – does not believe in this innovation, then they will not promote it: ‘*A long time ago, we had a dermatologist, who must have retired since. She was a chief physician who didn’t believe in it. The result was that we did very few, if any, PET scans for melanoma for six or seven years, even though it’s one of the main indications*’.

Our interviews similarly highlighted the importance of involving nuclear physicians in multidisciplinary team meetings (MDTs), as their participation helps to accelerate the spread of PET-CT. In this respect, MDTs are an essential forum for legitimizing this technology. As one oncologist emphasized: ‘*It was really through discussions with doctors from the nuclear medicine department during MDTs that I became aware of this surge in nuclear medicine*’. Interviews with nuclear medicine physicians confirm this observation and show that steps are proactively being taken in this direction, although the physicians have to deal with considerable constraints in terms of logistics and schedules. For example, one radiologist told us: ‘*We didn’t have a nuclear physician at every thoracic MDT. One would attend from time to time—whenever they could, really. They didn’t have time for that, or for a schedule that planned for a nuclear physician to systematically go to thorax*’.

## 4 Discussion

Our study analyzed the complex diffusion of PET-CT in France using a phased, mixed-methods approach. The initial quantitative analysis confirmed that while the supply of equipment and medical resources has expanded coherently across the country, the technology’s actual use—measured by the number of examinations—varies significantly between regions. To explain this disparity, our subsequent qualitative analysis of interviews with key actors identified a range of contextual factors at play. Moving beyond a simple description of the results, this section proposes several lines of analysis, particularly using the theoretical perspective of distributed governance and its associated “escape mechanisms,” to elucidate how and why these local dynamics can override national-level planning.

Our findings strongly support various studies and theoretical frameworks showing that the diffusion of innovative technologies is a complex and non-linear process, which depends on multiple contextual and non-technical factors (2, 6, 11). Even when innovations are based on sound scientific evidence, implementing them can still be a challenge (1, 12). The case of PET-CT fits into this analytical model, moving beyond a simple supply–demand logic to highlight the complexity and challenges of disseminating an innovative technology within established professional and institutional contexts. Our findings show that, in France, PET-CT technology was implemented gradually at first. While its dissemination subsequently accelerated, significant regional disparities have continued to exist, the specific impact of which on patient outcomes and health equity requires further investigation. In its 2016 report on medical imaging devices (MRI and CTscanners), the Cour des Comptes highlighted very significant differences in productivity from one machine to another, suggesting that, in addition to the number of machines installed, attention should be paid to the ways in which they are used. Our study shows that these findings apply to PET-CT.

Our results indicate that the regional disparities observed stem from the interaction of multiple factors operating at different levels of analysis. While some of these factors have already been explored in the literature, our study highlights several of them as having a significant influence on the specific dynamics of PET-CT use. The rapid rise of nuclear medicine (NM) is a constant challenge for it, as it entails collaboration with other specialties. In this respect, the introduction of PET-CT is testing and pushing NM physicians’ ability to cooperate with the requesters on whom they depend (13). The field of oncology has gradually been structured around several disciplines. While certain professionals, such as surgeons and radiotherapists, are in charge of the overall treatment, other professionals, like pathologists (14), geneticists, geriatricians, radiologists, or NM physicians, are involved only at a certain point in the diagnosis, treatment and follow-up of cancer patients. Innovations must contend with established clinical practices. The introduction of an innovation such as PET-CT redefines the outlines of professional skills and transforms professional relations and the division of labor (15, 16).

Another learning from this research concerns the influence of local contexts on PET-CT dissemination dynamics. While the literature largely highlights factors such as the availability of medical and paramedical resources and specialist skills (2), our results suggest that, rather than a mere matter of resources, these regional disparities are also the result of structural and organizational choices in terms of healthcare provision. Hospital departments’ organization models play a decisive role in the adoption and use of PET-CT. In this respect, our findings are in line with the work of Bucher (17) on the differentiation of professional segments, with some leaning towards scientific research and others towards clinical practice. Our study shows that some establishments are more firmly rooted in a scientific and academic approach, leading to differences in the intensity of PET-CT use. Beyond this distinction, the organization of services is another factor in regional variability in the use of PET-CT. As Denis et al. (12) pointed out, the adoption of a technology does not necessarily guarantee its optimal use, which is strongly influenced by structural factors such as resource planning, scheduling, and integration into existing care pathways. Our results confirm this perspective by showing that differences in PET-CT usage are not solely due to the number of devices available, but also to organizational factors such as service opening hours, interdisciplinary coordination, and the flexibility of hospital structures to absorb growing demand.

Finally, one last factor that may explain the dynamics around PET-CT is technological complementarity. The rise of PET-CT is closely linked to nuclear medicine’s ability to innovate in the field of radiopharmaceutical drugs. As new medical applications for PET-CT are discovered, the technology becomes more widespread and more useful. This dynamic coupling of ‘technology and radiopharmaceuticals’ has made PET-CT more attractive to requesters. In the field of medical innovation, a dynamic process can be set in motion: the more a technology is adopted, the more attractive it becomes. PET-CT adoption has been therefore bolstered by what Arthur (18, 19) calls ‘self-reinforcing mechanisms’, including “informational increasing returns”. This framework helps explain how early, context-driven advantages in certain regions became entrenched over time: the more a technology is adopted and discussed in forums like multidisciplinary teams (MDTs), the more attractive it becomes, widening the gap with regions that had slower initial uptake. Multidisciplinary team meetings are places where the knowledge, perspectives and concerns of multiple players come together to participate in the development of innovative diagnostic and therapeutic strategies (13). In oncology, they are an object for standardizing patient care (20, 21). However, this dynamic relies on a long learning process that can be slowed down by the lack of available medical resources, as nuclear medicine is a small specialty.

Our study does have several significant limitations, beyond simple data collection. Methodologically, our quantitative indicators for ‘supply’ and ‘demand’ are aggregate proxies; the demand indicator, in particular, lacks the granularity to account for varied imaging requirements across different cancer types. Concurrently, our key metric in Figure 2 (PET/incidence ratio) relies on a specific operationalization that impacts its interpretation. In France, most common cancers (breast, prostate, colorectal) have few geographical differences in incidence and the distribution of incidence and mortality for many cancers reflects well-known risk factors (smoking and alcohol consumption). However, the spatial distribution of mortality from these cancers is more heterogeneous, with excess mortality in the northern part of the country for women (breast and colorectal) and in the northern and central parts for men (prostate and colorectal). Moreover, geographical disparities exist for certain cancers (skin, melanoma, central nervous system, thyroid, and multiple myeloma). For instance, there is an excess of incidence and/or mortality for prostate, cervical, and stomach cancers, as well as multiple myeloma in the French Antilles (Guadeloupe and Martinique). As France exhibits regional heterogeneity in cancer incidence profiles—with some regions displaying disproportionate rates of PET-CT-indicated cancers—our aggregate demand proxy (total cancer prevalence) may introduce a systematic bias into the PET/incidence ratio displayed in Figure 2. Regions with a higher structural prevalence of PET-CT-indicated cancer types would be expected to show higher ratios independent of organizational performance. Future research should stratify demand indicators by cancer type to produce more granular regional benchmarks.

Furthermore, our qualitative findings on organizational dynamics, while illustrative, are based on a purposive, institution-focused sample and cannot be generalized to the entire French system. This sample bias also means the crucial perspectives of patients and communities are absent. Finally, the study’s context—a mature technology within the French healthcare system—limits the generalizability of our findings to emerging innovations like AI or to other regulatory structures.

The most profound limitations are conceptual. First, our analysis identifies regional disparities but lacks a deep “equity analysis” required to understand the socioeconomic impacts. Our macro-level data cannot determine who—based on income, education, or urban–rural geography—faces compounded barriers to access, even within well-equipped regions. Future research must move beyond asking “where” the technology is to “who” can actually access it and why some patient groups may be implicitly constructed as ‘non-users’. Second, this study does not forge a “stronger link between diffusion patterns and health outcomes”. We have successfully mapped “diffusion” (the spread) but not “dissemination”—what Berwick defines as the successful adoption of an innovation that actually leads to better care (22). We do not know if high-utilization regions are achieving better cancer survival rates, earlier-stage diagnoses, or lower long-term costs. Quantifying this gap between technology deployment and genuine, equitable patient value remains an important next step for policymakers.

While innovation literature often isolates diffusion factors, our study proposes an integrated, multi-level reading. We argue that the regional disparities we observed are a direct consequence of France’s move toward *distributed governance*. This model, marked by the decentralization of decision-making (e.g., to the ARS) and the empowerment of professionals, is intended to increase local flexibility. However, it paradoxically introduces “escape mechanisms” by fragmenting responsibility and weakening overall coordination. The case of PET-CT perfectly illustrates this tension: its diffusion was not a linear, rational deployment based on technological effectiveness but was instead shaped by these local escape mechanisms, leading to the uneven trajectories and utilization our data reveals.

## 5 Conclusion

Despite ongoing efforts to reduce variation in medical decisions and practices (guidelines, standardization of training, consensus conferences, etc.), the diffusion of technological innovations remains uneven, what must be presumed to be severe and inequitable consequences for patients who may not have access to the most effective and up-to-date care strategies. This is not merely a logistical failure but a profound equity failure. Our findings converge with those of many other studies showing that innovations never spread uniformly across healthcare systems (15) but add urgency by linking this non-spread to specific organizational and professional dynamics that can be addressed. This unevenness has potentially harmful consequences for patients who may not have access to the most effective and up-to-date care strategies, an issue of health equity that warrants urgent attention.

This observation calls for analyses of technological dissemination policies to be broadened beyond quantitative criteria alone. It is no longer sufficient to incorporate a ‘detailed reflection’; future policies must be explicitly designed to overcome the socioeconomic and structural barriers that our regional analysis implies, recognizing that different patient groups must be actively ‘constructed’ as users. Furthermore, these policies must be measured not by adoption rates, but by their direct, measurable impact on patient health outcomes by incorporating a more detailed reflection on models of healthcare provision and the local conditions under which medical innovations are appropriated. In this paper, we have presented an overview of the diffusion of PET-CT innovation in France. With the rapid emergence of therapeutic innovations in nuclear oncology, understanding contextual determinants is becoming an urgent issue for healthcare provision.

## Data Availability

The raw data supporting the conclusions of this article will be made available by the authors, without undue reservation.
